# Data visualization of complex research systems aligned with the sustainable development goals

**DOI:** 10.3389/fdata.2025.1562557

**Published:** 2025-06-09

**Authors:** Francisco Carlos Paletta, Audilio Gonzalez-Aguilar, Lise Verlaet

**Affiliations:** ^1^Universidade de São Paulo, Escola de Comunicações e Artes, São Paulo, Brazil; ^2^Groupe Laboratoire Paragraphe (EA 349), Université Paris 8, Saint-Denis, France; ^3^Groupe LHUMAIN Université Paul-Valéry Montpellier III, Montpellier, France

**Keywords:** sustainable development goals, complex systems, data visualization, network analysis, CIRAD

## Abstract

This study presents a methodological framework for visualizing the alignment between complex research systems and the Sustainable Development Goals (SDGs), using CIRAD as a case study. By leveraging advanced data visualization and bibliometric analysis, the research maps CIRAD's publications to the SDGs and explores thematic priorities and institutional collaborations. The findings underscore CIRAD's significant contributions to climate action, food security, biodiversity conservation, and rural development. The integration of complex systems theory and network analysis enhances understanding of SDG interlinkages and provides actionable insights for strategic decision-making in research governance.

## 1 Introduction

The United Nations' 2030 Agenda for Sustainable Development established 17 SDGs to address the world's most urgent challenges, emphasizing the need for coordinated efforts across various sectors, including agriculture, energy, transport, and health.

Research institutions are strategic players in generating knowledge, technologies, and evidence-based solutions that can accelerate sustainable development. Their contributions are key for creating policies and practices that align with the SDGs.

Assessing the alignment of large, decentralized research systems with the SDGs presents significant methodological challenges due to the interdisciplinary nature and scale of these systems. This study aims to address these challenges by proposing a methodology to map research outputs to the SDGs using data visualization techniques.

Previous studies have highlighted the complexity of research systems and the importance of data visualization in understanding and advancing SDGs. For instance, network theory and resilience theory provide tools to analyze the structure and dynamics of interconnected systems, revealing critical actors and potential points of intervention.

The main objectives of this study are to analyze CIRAD's research publications to understand their contribution to various SDGs, particularly those related to climate action, life on land, and sustainable agriculture. The research gap addressed by this study is the lack of a comprehensive methodology for mapping research outputs to the SDGs, which can provide valuable insights for evidence-based decision-making.

One approach is to use academic publications as tangible results that provide insight into the focus and priorities of research institutions. Mapping academic publications to topics related to the SDGs provides a consistent basis for assessing how research activities align with the SDG framework. It also makes it possible to understand linkages between the goals that arise from the co-occurrence of publications. Comparative analysis can also reveal differences in alignment with SDG SDGs between departments within the same institution. In this way, mapping research publications for the SDGs helps to map guidelines and networks within complex research systems.

This study proposes a methodology to map research outputs to the SDGs using data visualization techniques, focusing on the case of CIRAD, a French agricultural research organization. The approach involves analyzing CIRAD's research publications to understand how they contribute to various SDGs, particularly those related to climate action (SDG 13), life on land (SDG 15), and sustainable agriculture (SDG 2). By leveraging tools such as Gephi for network analysis and visualization, this study aims to uncover patterns, thematic priorities, and collaboration networks within CIRAD's research activities.

The main research questions guiding this study are: How does CIRAD's research align with the Sustainable Development Goals? Which SDGs receive the most focus in CIRAD's research outputs? What insights can data visualization provide about the relationships between different SDGs in CIRAD's research?

The key contributions of the study are: A comprehensive methodology for mapping publications to SDGs using advanced data visualization techniques. Insights into CIRAD's research focus and its contributions to specific SDGs, providing a model that other institutions can adopt. Policy recommendations for enhancing the alignment of research outputs with the SDGs through targeted funding and interdisciplinary collaboration.

The study was conducted to investigate how complex research systems can be effectively aligned with the Sustainable Development Goals (SDGs) using data visualization techniques. The main purpose of the study was to assess CIRAD's contribution to sustainable development by analyzing its research activities about the SDGs. Specifically, the study aimed to answer the following question: How does CIRAD's research contribute to the achievement of the SDGs, and which specific goals are most supported by its work?

The Sustainable Development Goals (SDGs) represent a global call to action to address environmental protection, climate change, poverty eradication, and the enhancement of quality of life for all (Mishra et al., 2024).

This study presents a method for linking research publications to the SDGs. For this purpose, the institutional repository of CIRAD, a French agricultural research organization, is used. The network analysis visually represents the links between the SDGs based on the CIRAD publication mappings. A comparative analysis shows the differences in the weighting of the different SDGs between CIRAD departments. The methodology consists of a universally applicable approach to systematically track, assess, and strengthen the alignment of complex systems of research organizations with the SDGs. Mapping scientific publications around the SDGs provides valuable insights into the structure and focus of different research activities. The technique can thus help institutions assess their alignment with the SDGs and steer their research activities.

Data visualization plays a strategic role in understanding the complexity associated with aligning large research systems like CIRAD with SDGs. By visualizing research activities, it becomes possible to identify trends, patterns, and connections that would otherwise remain hidden, providing a clearer picture of CI-RAD's contributions. The use of visualization tools enables a more accessible and systematic assessment of the alignment of research results with different sustainability goals, which supports evidence-based decision-making and increases transparency. This context underscores the value of visualizing complex research systems to improve their alignment with the global Sustainable Development Goals.

## 2 CIRAD's contribution to the sustainable development goals: a theoretical and applied analysis

**Figure 1:**
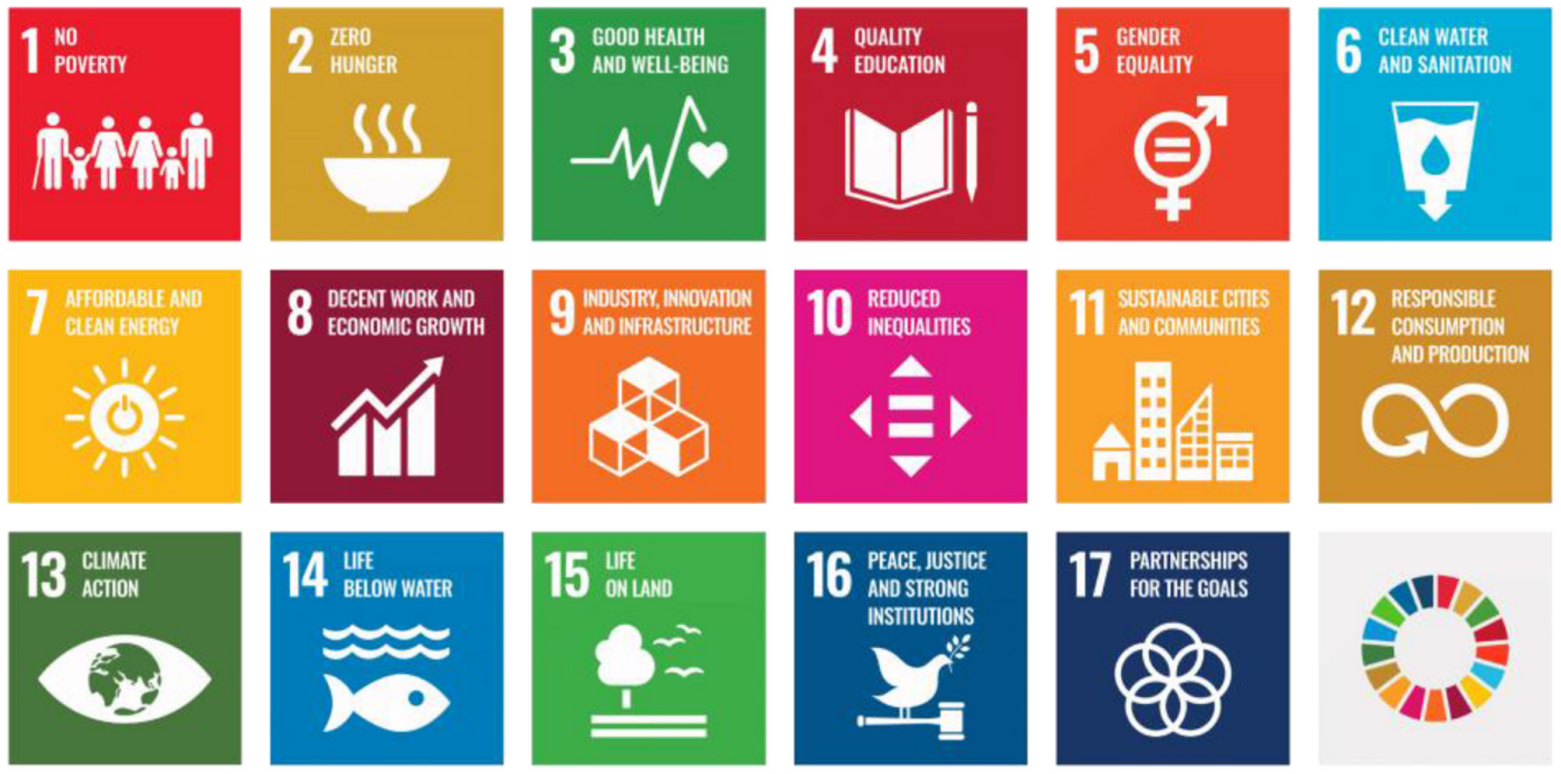
Sustainable development goals.

CIRAD is the French agricultural research and cooperation organization working for the sustainable development of tropical and Mediterranean regions and constitutes a paradigmatic case of how research institutions can strategically align their agendas with the United Nations' 2030 Agenda for Sustainable Development. Through a systemic, interdisciplinary, and impact-oriented research approach, CIRAD contributes to addressing the interdependent challenges embedded in the 17 Sustainable Development Goals (SDGs) (United Nations, 2015). The 17 SDGs is shown in Figure 1.

This figure presents the 17 Sustainable Development Goals (SDGs) established by the United Nations as part of the 2030 Agenda. It highlights the interconnected nature of these goals, emphasizing that achieving one often contributes to others. For example, promoting renewable energy (SDG 7) helps mitigate greenhouse gas emissions (SDG 13) while also improving health (SDG 3) and food security (SDG 2). The figure underscores the need for holistic approaches to sustainable development that address root causes and promote synergies among different goals.

The SDGs form an integrated framework that addresses complex global issues, especially at the intersection of agriculture, environmental sustainability, and social equity. CIRAD's research focuses—including climate action (SDG 13), life on land (SDG 15), zero hunger (SDG 2), no poverty (SDG 1), gender equality (SDG 5), and inclusive economic growth (SDG 8)—illustrate a comprehensive orientation toward multiple development objectives. The institution has pioneered interdisciplinary methodologies that integrate sustainable agricultural practices, agroecological systems, biodiversity conservation, and technological innovation to advance long-term development outcomes (FAO, IFAD, UNICEF, WFP and WHO, 2024; CIRAD, 2023).

**Figure 2:**
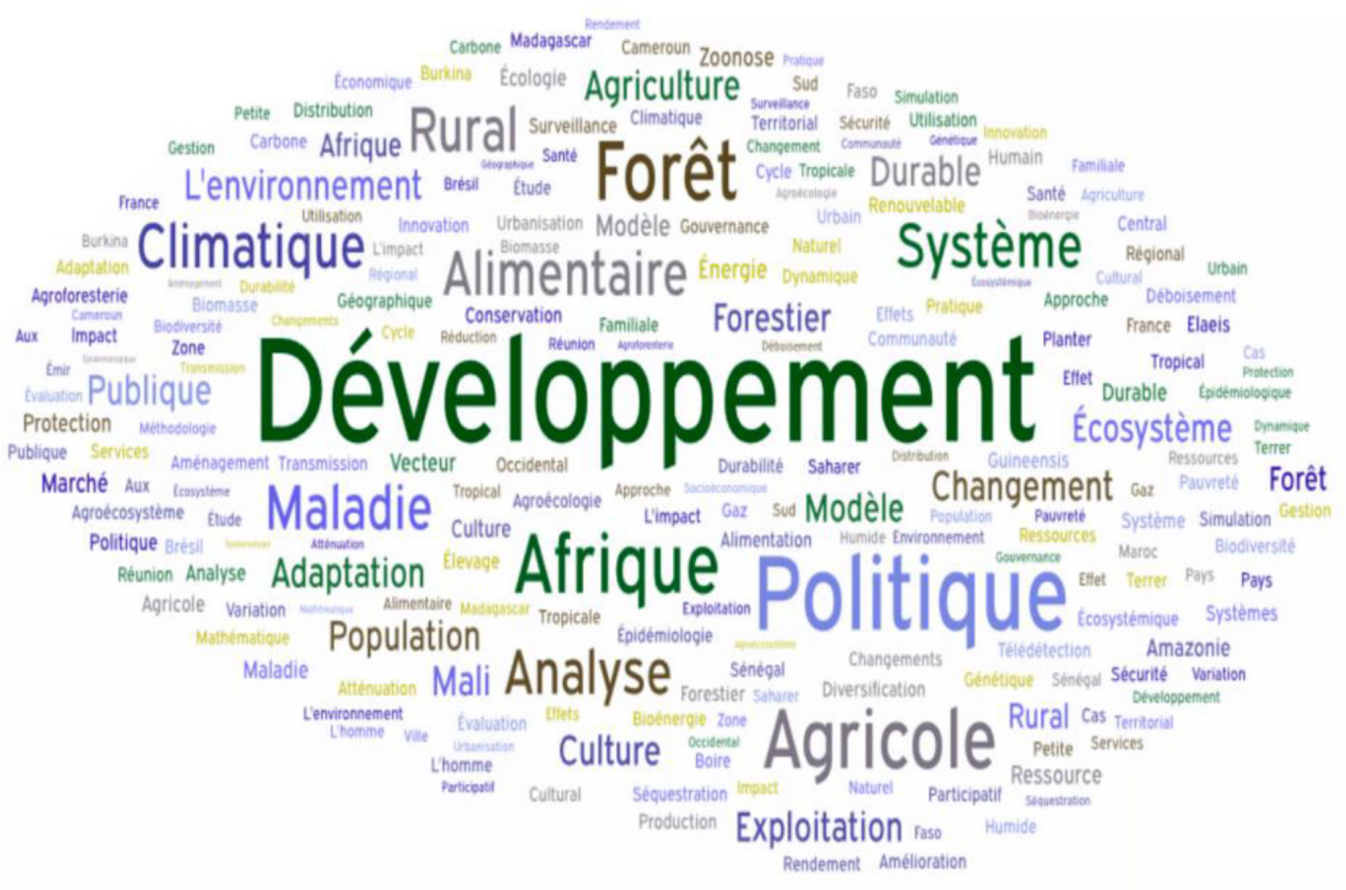
CIRAD publications keyword cloud.

Beyond thematic alignment, CIRAD's research is grounded in a theoretical understanding of complex systems. Theoretical frameworks such as network theory, resilience thinking, and systems dynamics underpin CIRAD's approach to mapping interactions across the SDG ecosystem (Le Blanc, 2015; Weber et al., 2021). These frameworks allow for a nuanced analysis of feedback loops, co-benefits, and trade-offs across development goals—thus enabling CIRAD to identify leverage points for transformative change (Figure 2).

CIRAD operationalizes these frameworks through integrative research that yields cross-cutting benefits. For example, its agroecology programs contribute to SDG 2 (Zero Hunger) by enhancing food system resilience, while simultaneously supporting SDGs 13 and 15 through improved carbon sequestration and land stewardship (Harfouche et al., 2024). Likewise, its gender-inclusive rural development initiatives demonstrate alignment with SDG 5 by promoting equity in access to resources and decision-making structures (Mishra et al., 2024).

In parallel, CIRAD engages in transnational cooperation and knowledge transfer, particularly with countries in the Global South, reflecting a deep commitment to SDG 17 (Partnerships for the Goals). Such partnerships are not only instrumental in strengthening local capacities but also foster epistemic diversity in sustainable development research (Sachs et al., 2019).

Recognizing the methodological complexity of mapping scientific outputs to specific SDGs, CIRAD leverages advanced data visualization and bibliometric tools. Techniques such as Social Network Analysis (SNA) and keyword mapping enable a clearer understanding of collaboration networks, research clusters, and thematic priorities (Andrienko et al., 2020). These tools help bridge the gap between abstract policy frameworks and measurable research contributions, providing stakeholders with actionable insights for decision-making.

Overall, CIRAD illustrates how research institutions can serve as both epistemic and operational agents of sustainable development. Its approach demonstrates the value of embedding systems, thinking into applied research and policy frameworks. In doing so, CIRAD offers a replicable model for aligning scientific inquiry with the integrated logic of the SDGs, and for enhancing institutional capacity to contribute meaningfully to global sustainability transitions.

## 3 Methodological approaches to addressing complex systems in SDG-related research

This methodological approach focuses on the visualization of complex systems related to Sustainable Development Goals (SDGs) research. By leveraging advanced data visualization techniques, this study aims to provide a clear understanding of the intricate relationships, collaboration networks, and thematic priorities within SDG-related research, contributing to a better understanding of the dynamics involved in sustainable development.

The use of advanced search functionalities and data extraction techniques enables the identification and collection of relevant information from Agritrop databases, facilitating an in-depth exploration of research trends and collaboration networks. These data are then visualized using advanced tools such as Gephi, Cosma, Cytoscape, and Tableau, which transform complex datasets into visually accessible representations that highlight key players, research clusters, and emerging themes.

Visualization plays an important role in communicating the impact of research activities by offering a clear and intuitive way to present relationships and patterns within the data. This allows researchers and stakeholders to explore the dynamics of scientific collaboration, understand knowledge exchange, and identify areas of strength and opportunities for development. Techniques such as network analysis and mapping methods allow hidden insights to be discovered, providing a deeper understanding of CIRAD's contributions to the SDGs and the overall impact of its research efforts (Centre for Complex Systems Modelling, 2018).

By creating informative and easy-to-interpret visual representations, this methodological approach supports evidence-based decision-making processes, empowering stakeholders, policymakers, and funding agencies to make informed decisions about resource allocation and strategic interventions. These visualizations make it possible to identify areas of competence and highlight opportunities to improve investments in SDG-related research (United Nations Sustainable Development Solutions Network, 2019).

Visualizing CIRAD's contribution to the SDGs helps to raise awareness among key stakeholders about the importance of investing in agricultural research and sustainable rural development. This approach not only enhances transparency and accountability but also fosters collaboration and coordination to address sustainable development challenges globally.

### 3.1 Social networks analysis (SNA) in SDG and complex systems research

Social network analysis (SNA) is a fundamental methodological tool in the visualization and study of complex systems, especially in research related to the SDGs. SNA allows for mapping and analyzing relationships and structures among actors within a network, identifying collaboration patterns, influence, and information flows that are essential for understanding how different components of a complex system interrelate (Wasserman and Faust, 1994).

In the context of SDG research, SNA facilitates the identification of key actors and their connections within scientific networks, helping to understand how research efforts are organized globally and how knowledge is shared. The application of SNA not only enables the visualization of collaborations between institutions and sectors but also identifies nodes and links that have the greatest impact on advancing sustainability goals.

The visualization of social networks through SNA also reveals communities and research clusters within the data, which is essential for identifying groups of experts working on specific SDG topics and determining how these groups interact. This is particularly useful for understanding the dynamics of international cooperation in agricultural research and other areas critical to sustainable development.

Using SNA together with visualization tools such as Gephi and Cytoscape allows researchers to transform large volumes of data into graphical representations that facilitate the interpretation and communication of complex results. These representations provide an intuitive view of the network structure, making it easier to make decisions about how to strengthen weak connections or reinforce strategic areas within the SDG research network.

The capabilities of SNA to unravel the inherent complexities of research systems also allow for assessing the cohesion and effectiveness of scientific networks. This is important to create policies and collaboration strategies that drive sustainable development, ensuring that resources are allocated effectively, and initiatives are directed toward maximum impact.

### 3.2 Data collection: Agritrop

In Data Collection, we utilized the Agritrop website (https://agritrop.cirad.fr) which provides access to a rich source of data covering various aspects of agricultural research and development.

This comprehensive dataset from the Agrotrop database allows us to explore SDG-related issues in depth, providing a holistic understanding of the interconnectedness of the Sustainable Development Goals. Using the Agritrop website as a comprehensive source of data related to our research we have processed and prepared the data to perform our network analyses.

In extracting the information from the Agritrop databases, we included the publications organized by each Sustainable Development Goal (SDG). Using search and export functionalities of the data in Zorero, Reference Manager (RIS) format, we retrieved datasets for all 17 Sustainable Development Goals (SDGs).

By creating a dashboard of the publications, we separated specific data based on keywords, abstracts, publication dates, authors, and thematic areas aligned with the SDGs from the publications and keywords into an Excel sheet. We have made a treatment to the keywords of lowercase transformation of all words, and deletion of all spaces to establish the relationship between the keywords and the 17 Sustainable Development Goals (SDGs).

From this Excel file, we have processed the 45,779 keywords to eliminate the repeated words and to be able to elaborate a table of unique keywords (5,638). From these two Excel tables, we created the nodes table and the links table to perform our network analysis with the Gephi software.

This comprehensive dataset allows for an in-depth exploration of topics relevant to the SDGs, providing a holistic understanding of the interconnection of agricultural systems with sustainable development goals. Data collection was conducted according to the methodological procedures presented in Table 1.

**Table 1:** Data collection methodology.

Database: the Agritrop website as a source of data related to agricultural research and development https://agritrop.cirad.fr/recherches_odd.html
Relevant information was extracted from Agritrop's databases, including publications, projects, and partnerships, focusing on topics relevant to the Sustainable Development Goals (SDGs).
Advanced search functionalities were applied in order to filter and retrieve specific data sets based on keywords, auto-res, publication dates and thematic areas aligned with the SDGs.
The integrity and quality of the data was guaranteed by cross-referencing information from Agritrop with other reliable sources and conducting data validation procedures.
Excel (dynamic graphics) was used for statistical analyses.

### 3.3 Visualization and mapping methods and tools

The methodological procedure used visualization techniques to represent and analyze the complex scientific networks related to the SDGs, derived from the data collected from Agritrop.

To visualize the relationships between researchers, institutions, and publications within the SDG research domain, the Gephi platform was used. Network analysis algorithms were applied to identify key players, influential research topics, and collaboration patterns within the SDG scientific community. Gephi was integrated with gexf.js to enhance visualization capabilities and generate interactive and dynamic visualizations of scientific networks related to the SDGs.

Advanced mapping methods were used to geographically visualize the distribution of research activities, funding sources, and impact metrics related to the SDGs in different regions and countries. Social Network Analysis (SNA) was used as a methodology to visualize the data. SNA utilizes network and graph theories (Andrienko et al., 2020). The software used to create these visualizations was Gephi (Bastian et al., 2009): https://gephi.org.

Gephi is a program for visualizing, exploring, and understanding all types of graphs and networks (Cherven, 2015). It is free and based on ARS. The spatialization algorithms used were Atlas Force 2 and Atlas 2-3D. It was combined with a visualizer called gexf.js that allows graphics created with Gephi to be exported to the web, available on Github: https://github.com/raphv/gexf-js

The final visualization is displayed as an interactive map that users can manipulate to analyze the results by applying different integrated filtering strategies. These tools allow for an intuitive understanding of scientific networks and promote more informed, data-driven decision-making, facilitating the identification of critical points and collaboration opportunities within the SDG context.

The application of SNA together with Gephi allows for transforming large volumes of data into clear and comprehensible graphical representations. These visualizations help identify the underlying structures and information flows that are part of the research networks on SDGs, providing a detailed view of international cooperation and the dynamics of complex systems in the realm of sustainable development (Andrienko et al., 2020).

## 4 Results and discussion

Agritrop, a bibliographic database developed by CIRAD (Centre de recherche agronomique pour le development), contains more than 1.5 million references covering topics related to tropical and Mediterranean agriculture. The analysis of these references provides valuable insights into CIRAD's research priorities, especially regarding the alignment of its research outputs with the Sustainable Development Goals (SDGs).

### 4.1 Quantitative analysis of the distribution of publications by SDGs

Table 2 presents the distribution of CIRAD's publications across different SDGs. The quantitative analysis reveals that Goal 13: Climate Action and Goal 15: Life on Land has the highest number of publications, with 2,191 and 938, respectively, together representing ~59% of CIRAD's total publications related to the SDGs. This focus underscores CIRAD's commitment to addressing climate change and promoting the sustainable management of terrestrial ecosystems. In contrast, Goal 16: Peace, Justice and Strong Institutions and Goal 17: Partnerships for the Goals have the lowest number of publications, with only 21 and 43, respectively.

**Table 2:** Number of publications per sustainable development goal.

**SDGs**	**No. publication**	**Unique keywords**	**Keywords-total**
GOAL 13	2,191	2,796	17,261
GOAL 15	938	110	8,941
GOAL 2	367	865	3,893
GOAL 3	307	1,078	3,332
GOAL 11	386	70	2,915
GOAL 12	293	66	2,160
GOAL 7	240	58	1,833
GOAL 1	87	399	1,043
GOAL 5	125	47	1,008
GOAL 8	63	23	642
GOAL 17	43	11	565
GOAL 9	57	16	553
GOAL 6	54	26	434
GOAL 10	53	25	405
GOAL 14	42	22	399
GOAL 4	40	17	230
GOAL 16	21	9	165
Total	5,307	5,638	45,779

This distribution highlights CIRAD's emphasis on topics such as climate resilience, biodiversity conservation, and sustainable agricultural practices, which are needed for achieving food security and mitigating the impact of climate change. Conversely, the small number of publications on Goals 16 and 17 suggests that CIRAD may have fewer ongoing initiatives related to governance, institutional capacity, or cross-sectoral partnerships, Table 3.

**Table 3:** CIRAD publications keyword cloud.

**Word**	**TF-IDF**	**Weight**
climate change	0.019	855
biodiversity	0.016	752
food security	0.014	619
France	0.012	549
adaptation to climate change	0.009	438
Environmental impact	0.008	353
Use of land	0.007	327
Natural Resource Management	0.007	326
Development policy	0.007	307
sustainable development	0.007	299
Tropical forest	0.006	267
Brazil	0.006	255
ecosystem services	0.005	250
Cropping system	0.005	242
agroecology	0.005	241
Case Study	0.004	203
agroforestry	0.004	201
Environmental policy	0.004	197
Impact assessment	0.004	197
sustainable agriculture	0.004	195
Africa	0.004	195
Carbon sequestration	0.004	192
deforestation	0.004	190
Agricultural development	0.004	189

**Figure 3:**
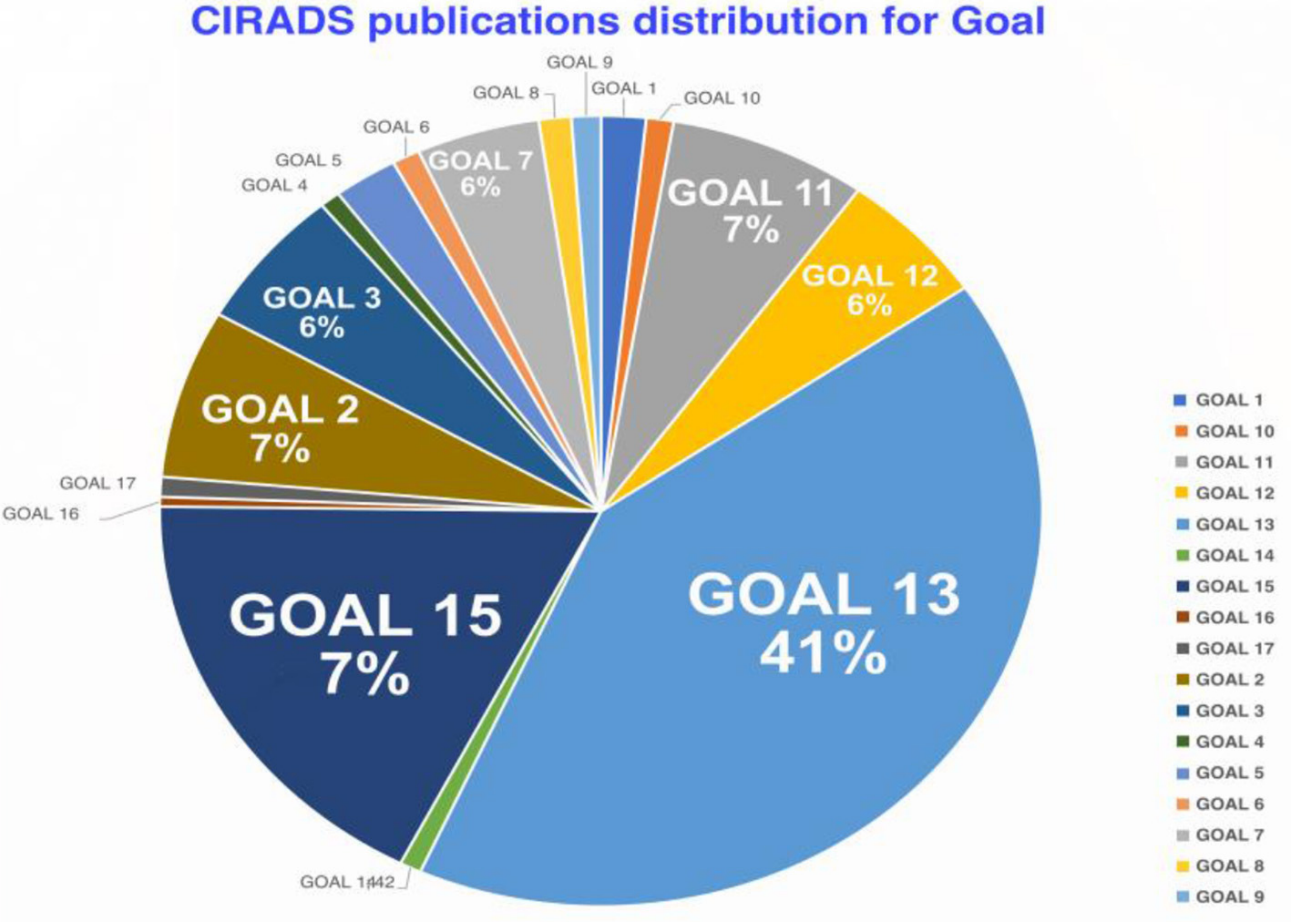
CIRADS publications distribution for goal.

The results, as shown in Table 3 and Figure 3, also include the Keyword Cloud based on the term frequency-inverse document frequency (TF-IDF) scores of keywords related to CIRAD's publications. Key themes such as “climate change,” “biodiversity,” “food security,” and “adaptation to climate change” were found to be the most frequently used terms, indicating a strong focus on environmental sustainability and climate resilience in CIRAD's research.

This keyword cloud visualizes the most frequently used terms in CIRAD's research publications, using a term frequency-inverse document frequency (TF-IDF) approach. Prominent keywords include “climate change,” “biodiversity,” and “food security,” indicating CIRAD's research focus on environmental sustainability and resilience. The size of each keyword reflects its frequency and relevance, providing a clear visual summary of CIRAD's thematic priorities.

This chart displays the distribution of CIRAD's publications across various SDGs. The figure shows that SDG 13 (Climate Action) and SDG 15 (Life on Land) have the highest number of publications, highlighting CIRAD's emphasis on addressing climate change and promoting sustainable land management. In contrast, SDG 16 (Peace, Justice, and Strong Institutions) and SDG 17 (Partnerships for the Goals) have the fewest publications, suggesting potential areas for strategic expansion.

**Figure 4:**
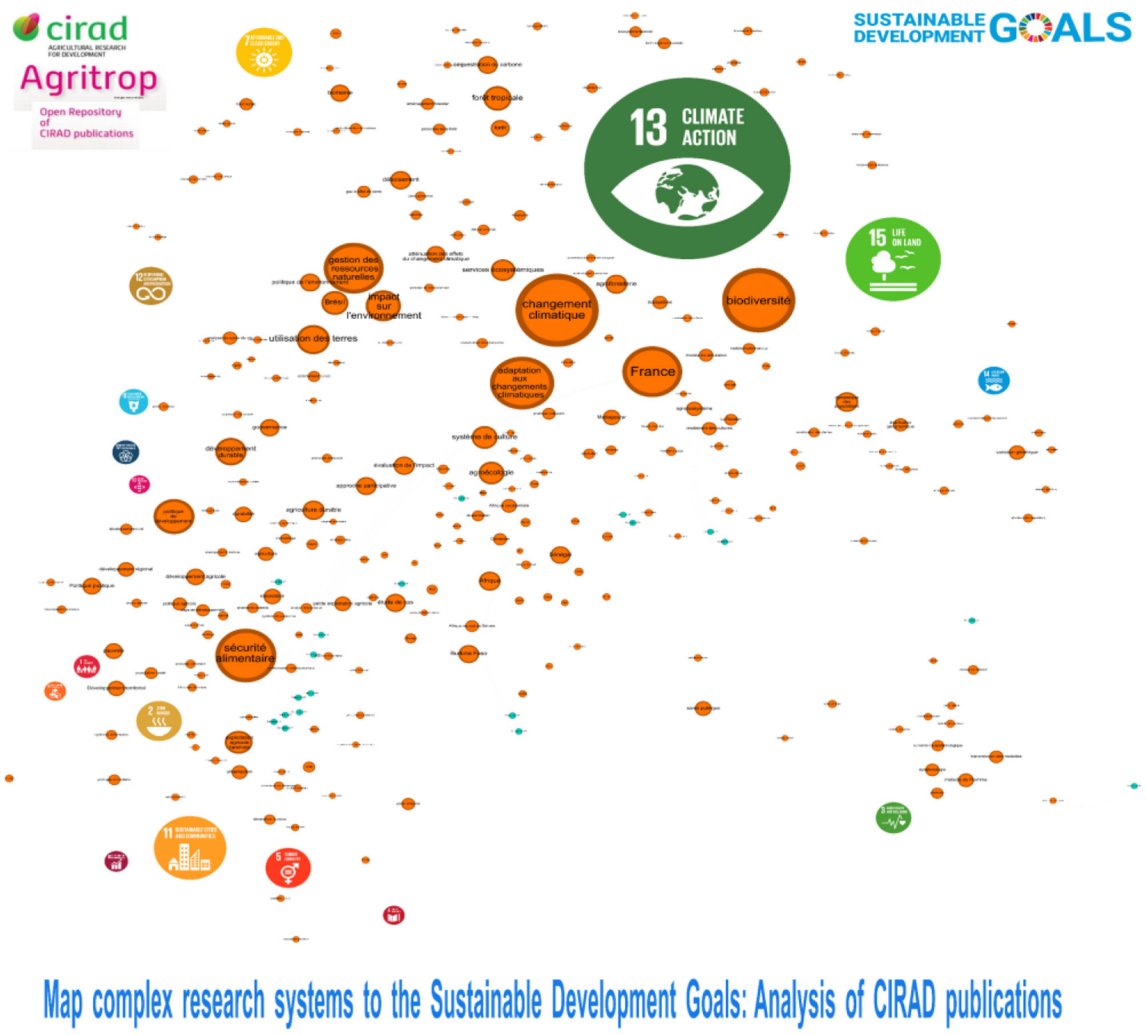
Mapping of Cirad (Agritrop) publications 2016–2023. Interact Version (https://metroteach.com/SDG/index.html.) Network Properties.

Figure 4 shows the overall distribution of publications by SDGs, highlighting CIRAD's strategic alignment with specific sustainable development objectives. The predominance of SDG 13 and SDG 15 illustrates CIRAD's dedication to contributing to global climate action and land management, which are both pressing issues in the context of global climate change and natural resource management.

**Figure 5:**
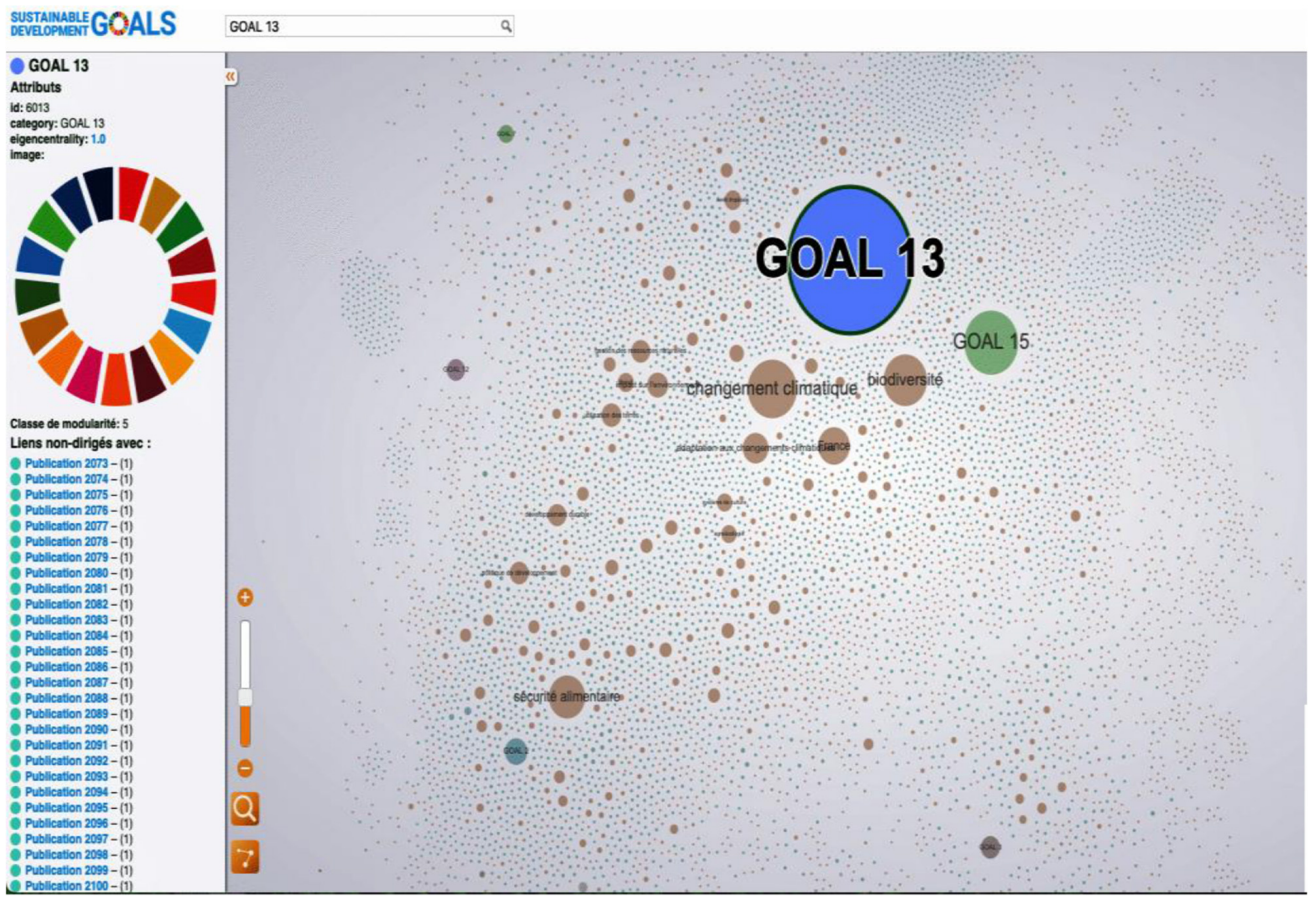
Goal 13—interactive tool allows you to explore CIRAD publications (http://metroteach.com/SDG/index.html).

The quantitative data is complemented by network analysis, as shown in Figure 5. The mapping of CIRAD's publications from 2016 to 2023 reveals a complex network with 9,812 nodes and 45,580 links, indicating extensive collaboration among researchers, institutions, and topics. The network properties, including a density of 0.001 and a modularity of 0.459, suggest that while the network is sparsely connected, it exhibits distinct clusters or communities that reflect specialized areas of research focus.

- Number of nodes: 9,812.
- Number of links: 48,580.
- Density: 0.001 (very sparsely connected).
- Eigenvector Centrality: 0.1062 (high value).
- Modularity: 0.459 (high modularity).

The eigenvector centrality value of 0.1062 further indicates that certain nodes in the network (i.e., certain researchers or topics) have considerable influence within the overall structure. This measure is critical for understanding which elements are central to CIRAD's SDG-related research, potentially guiding future collaboration and funding efforts, Figure 5.

This network map illustrates the relationships between different SDGs based on CIRAD's research outputs. It consists of 9,812 nodes and 45,580 links, indicating a complex network of collaborations among researchers, institutions, and topics. The map reveals distinct clusters representing specialized areas of research focus. High eigenvector centrality values suggest that certain nodes (research topics or institutions) play a central role in CIRAD's SDG-related research. The modularity score indicates well-defined clusters, supporting targeted strategies for enhancing interdisciplinary collaboration.

Overall, the analysis provides a comprehensive overview of CIRAD's research output about the SDGs. The strong emphasis on climate action and life on land reflects CIRAD's commitment to tackling some of the most pressing challenges facing the global community today. By focusing on these areas, CIRAD is helping to foster resilience, promote biodiversity, and ensure sustainable development in vulnerable regions.

The interactive version of the mapping, available through Figure 5 at this link (https://metroteach.com/SDG/index.html), allows stakeholders to explore the dynamics of CIRAD's SDG-related publications in greater detail, providing a valuable tool for understanding collaboration patterns and identifying opportunities for strategic partnerships in sustainable development.

The mapping of CIRAD's publications from 2016 to 2023 reveals a complex network with 9,812 nodes and 45,580 links, indicating extensive collaboration among researchers, institutions, and topics. The network properties, including a density of 0.001 and a modularity of 0.459, suggest that while the network is sparsely connected, it exhibits distinct clusters or communities that reflect specialized areas of research focus.

The eigenvector centrality value of 0.1062 further indicates that certain nodes in the network (i.e., certain researchers or topics) have considerable influence within the overall structure. This measure is critical for understanding which elements are central to CIRAD's SDG-related research, potentially guiding future collaboration and funding efforts.

Overall, the analysis provides a comprehensive overview of CIRAD's research output about the SDGs. The strong emphasis on climate action and life on land reflects CIRAD's commitment to tackling some of the most pressing challenges facing the global community today. By focusing on these areas, CIRAD is helping to foster resilience, promote biodiversity, and ensure sustainable development in vulnerable regions.

The interactive version of the mapping, available through Figure 5 at this link, allows stakeholders to explore the dynamics of CIRAD's SDG-related publications in greater detail, providing a valuable tool for understanding collaboration patterns and identifying opportunities for strategic partnerships in sustainable development.

**Figure 6:**
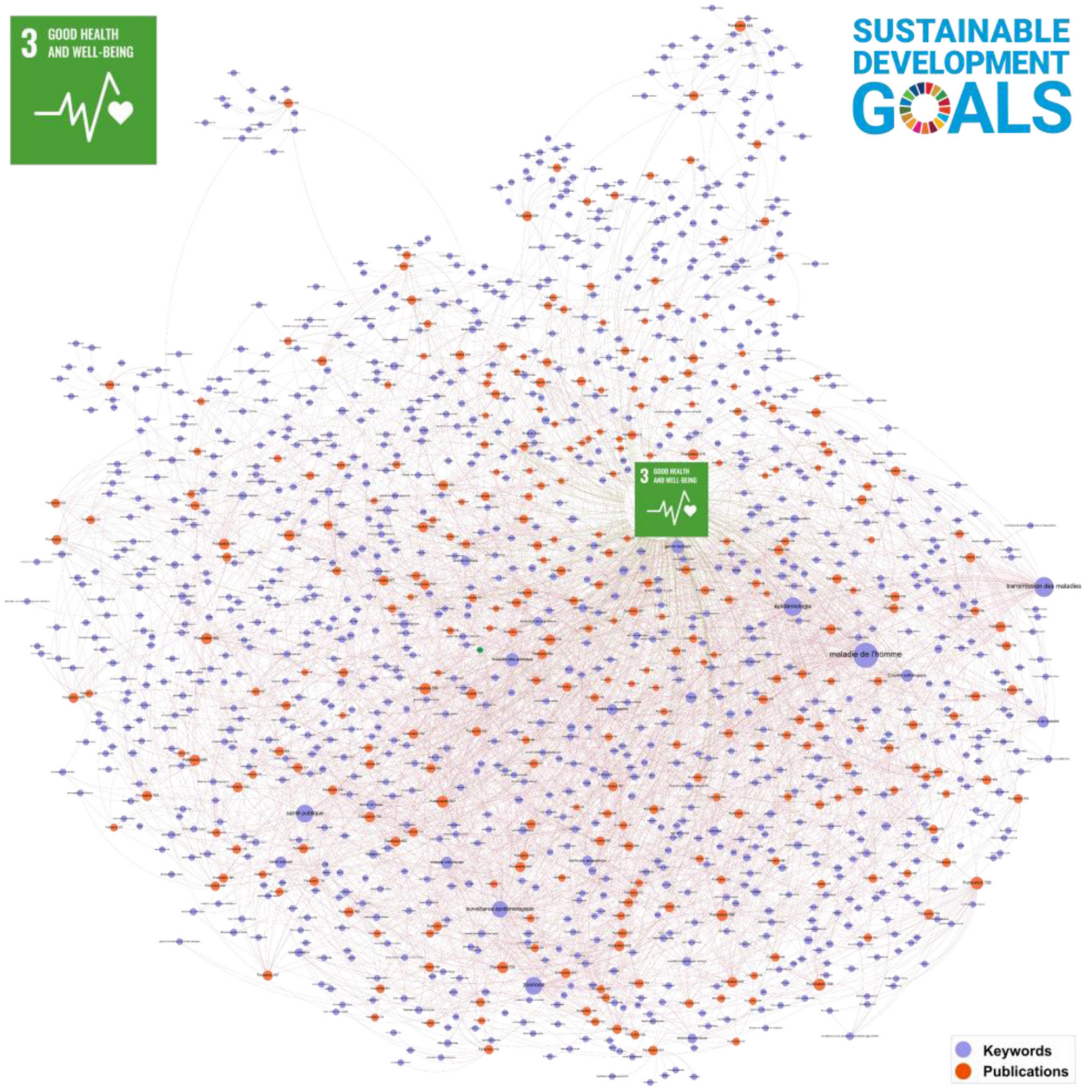
Goal 3—interactive tool allows you to explore CIRAD publications (http://metroteach.com/SDG/index.html).

Interpretation of Properties:

- The extremely low density indicates that the network is extremely sparsely connected, meaning most nodes are not directly connected. The high Eigenvector Centrality value suggests that influence is concentrated on a small number of key nodes.
- The high modularity indicates that the network is divided into distinct groups (modules) with stronger links within modules than between them. Figures 5, 6.

This figure showcases an interactive tool that allows users to explore CIRAD's publications related to SDG 13 (Climate Action). The tool visualizes research outputs and their interconnections with other SDGs, enabling stakeholders to understand how CIRAD's climate-related research contributes to broader sustainable development efforts. The interactive aspect allows for the examination of specific publications, co-authorship networks, and thematic overlaps.

Like Figure 5, this figure presents an interactive tool focused on SDG 3 (Good Health and Wellbeing). It maps CIRAD's research contributions to health-related topics, highlighting connections to other SDGs such as SDG 2 (Zero Hunger) and SDG 6 (Clean Water and Sanitation). The tool's interactive features enable users to filter publications by keywords, authors, and year, providing a detailed view of CIRAD's impact on health and wellbeing through agricultural research.

## 5 Conclusion

The analysis presented in this article highlights CIRAD's substantial contribution to the Sustainable Development Goals (SDGs) through its extensive research and publications. By leveraging the Agritrop database, we were able to visualize and assess CIRAD's alignment with the SDGs, with a notable focus on Goal 13: Climate Action and Goal 15: Life on Land, which collectively represent 60% of CIRAD's publications. This strong emphasis demonstrates CIRAD's commitment to address climate change, biodiversity conservation, and sustainable agricultural practices. The network analysis provided further insights into the structure and dynamics of CIRAD's research activities, revealing a complex network of collaborations among researchers, institutions, and topics. The high modularity value indicates that CIRAD's research is well-organized into distinct clusters, allowing for specialized research that effectively addresses specific areas of sustainable development. However, the low network density suggests opportunities for fostering greater interdisciplinary collaboration, which could lead to more integrated and impactful solutions to the challenges of sustainable development.

The interactive visualizations developed in this study offer stakeholders an effective tool for exploring the dynamics of CIRAD's SDG-related research, identifying influential nodes, and uncovering areas for strategic partnerships. This aligns with CIRAD's overall mission to promote agricultural sustainability, enhance food security, and support socio-economic development in vulnerable regions across the globe (World Bank Group, 2019).

This study presents a robust methodology for mapping research contributions to the Sustainable Development Goals (SDGs) through the integration of bibliometric analysis, social network analysis, and advanced data visualization techniques. The application of this framework to CIRAD's research ecosystem reveals significant alignment with key sustainability goals, most notably SDG 13 (Climate Action), SDG 15 (Life on Land), and SDG 2 (Zero Hunger)—highlighting the institution's strategic focus on climate resilience, biodiversity conservation, and sustainable agriculture.

The use of data from CIRAD's institutional repository (Agritrop) combined with analytical tools such as Gephi enabled the construction of an interactive research network, facilitating the identification of thematic priorities, collaboration patterns, and knowledge production dynamics. The network's structural metrics, including high modularity and low density, suggest the existence of distinct research clusters, offering opportunities for enhancing cross-cutting collaboration and interdisciplinary integration.

The methodological approach proposed herein is not only replicable but also scalable across diverse institutional and disciplinary contexts. Its successful application to CIRAD underscores its broader applicability for universities, public research organizations, international consortia, and specialized research centers. By aligning research outputs with SDG indicators, this approach enhances transparency, accountability, and strategic alignment with global development agendas (Table 4).

**Table 4:** Conclusion validity analysis.

**Applicability of research findings to other institutions and fields**
The methodology presented in this study, which maps research publications to the Sustainable Development Goals (SDGs) using advanced data visualization techniques, demonstrates significant potential for application across different research institutions and disciplines. By utilizing bibliometric analysis and visualization tools like Gephi, the approach allows for a comprehensive examination of research outputs, thematic priorities, and collaboration networks. Applicability in Other Research Institutions: Universities and Public Research Organizations: Institutions with diverse research agendas can adopt this methodology to assess their contributions to specific SDGs, identify gaps, and align their research priorities with global sustainability goals. International Research Consortia: Collaborative research networks can leverage the approach to visualize and coordinate efforts across institutions, enhancing synergy and impact toward the SDGs. Specialized Research Centers: Entities focusing on areas such as climate change, health, or renewable energy can benefit from the detailed mapping of research outputs to relevant SDGs, facilitating targeted funding and policy recommendations. Applicability Across Different Fields: Environmental Sciences: The methodology's emphasis on visualizing research impact aligns well with interdisciplinary studies on biodiversity, climate change (SDG 13), and water management (SDG 6). Social Sciences: By including indicators related to gender equality (SDG 5) and social justice, the approach can support institutions focusing on social policies and human rights. Agricultural and Food Sciences: The successful application of this methodology to CIRAD's research on food security (SDG 2) suggests its effectiveness for other agricultural research entities.
**Policy recommendations and practical insights**
Enhancing Research Visibility and Impact: • Policy Recommendation: Funding agencies and governments should encourage research institutions to adopt SDG-based mapping of publications to enhance visibility and demonstrate contributions to global goals. • Practical Insight: Establishing standardized protocols for categorizing research outputs based on SDGs would facilitate cross-institutional comparisons and collaborative opportunities. Promoting Interdisciplinary Collaboration: • Policy Recommendation: Develop funding mechanisms that incentivize interdisciplinary research projects explicitly aligned with SDGs, ensuring comprehensive and integrated approaches to complex global challenges. • Practical Insight: Visualization tools like Gephi can help identify potential collaborators by mapping co-authorship networks and thematic overlaps. Advancing Data-Driven Decision-Making: • Policy Recommendation: National and regional research policies should mandate the integration of bibliometric and visualization techniques for tracking research contributions to the SDGs, enhancing accountability and resource allocation. • Practical Insight: Research institutions can develop internal dashboards to monitor progress toward SDGs, using real-time data to adjust research strategies effectively. Addressing Challenges in SDG Classification: • Policy Recommendation: Create a task force of experts to refine SDG classification criteria for research outputs, reducing ambiguity and ensuring accurate mapping. • Practical Insight: Adopting natural language processing (NLP) techniques for text mining could enhance the precision of SDG mapping, particularly for publications with overlapping themes.

From a policy perspective, the findings offer actionable insights. Research institutions can strengthen the visibility and impact of their work through standardized SDG classification protocols and internal dashboards for monitoring progress. Policymakers and funding agencies are encouraged to adopt and incentivize such data-driven approaches to facilitate evidence-based decision-making, optimize resource allocation, and foster interdisciplinary collaboration aligned with the SDGs.

Ultimately, this study demonstrates that the mapping of research publications to the SDGs provides an effective instrument for understanding the contribution of research systems to sustainable development. By enabling the identification of synergies, gaps, and leverage points within complex networks, this methodology supports a more integrated and coherent research agenda—one that is capable of addressing the systemic challenges inherent to the 2030 Agenda.

In this context, it is important to assess the broader applicability of the proposed approach beyond the case study of CIRAD. Exploring its relevance to other research institutions and across various fields can deepen our understanding of the role of research in driving sustainable development.

Furthermore, translating these findings into actionable policy recommendations is essential to bridge the gap between research outputs and practical implementation. The following sections discuss the applicability of the methodology to other institutions and fields, followed by specific policy recommendations and practical insights that can support decision-makers in aligning research strategies with the SDGs.

In conclusion, CIRAD's research contributions significantly align with the global agenda of sustainable development, particularly in addressing climate action, biodiversity, and sustainable agriculture. By focusing on these critical areas, CIRAD continues to play an instrumental role in advancing global efforts toward the achievement of the SDGs, fostering resilience, and promoting a more sustainable future.

Considering the complex systems within the SDGs, mapping publications becomes even more relevant when considering the interconnected nature of the SDGs. By mapping publications on different SDGs, it is possible to identify synergies and potential conflicts between different SDGs, allowing for more holistic and integrated policy approaches as well as understanding the complex relationships between various SDG targets and the research efforts needed to achieve them. It is essential to promote transdisciplinary research that goes beyond disciplinary boundaries to address the interconnected dilemmas of the SDGs.

## Data Availability

The raw data supporting the conclusions of this article will be made available by the authors, without undue reservation.
